# Impact of different polishing techniques on surface roughness, gloss, and microhardness of zirconium oxide reinforced flowable bulk-fill resin composite: an in vitro study

**DOI:** 10.1186/s12903-025-06605-y

**Published:** 2025-07-26

**Authors:** Amr Elsayed Elnahas, Mohamed Elshirbeny Elawsya, Abeer ElSayed ElEmbaby

**Affiliations:** 1 Department of Conservative Dentistry, Faculty of Dentistry, Mansoura University, Algomhoria Street, P.O. Box 35516, Mansoura, Aldakhlia, Egypt; 2 Faculty of Dentistry, Mansoura National University, Gamasa, Egypt

**Keywords:** Composite resins, Dental polishing, Surface properties, Zirconium oxide

## Abstract

**Background:**

To evaluate the impact of different polishing systems on surface roughness (SR), surface gloss (SG), and vickers microhardness (VMH) of flowable bulk-fill composites reinforced with zirconium oxide fillers.

**Methods:**

Two flowable bulk-fill composites reinforced with zirconium oxide fillers (PALFIQUE BULK FLOW, Filtek Bulk Fill Flowable) and another one with conventional fillers (Tetric N-Flow Bulk Fill) were used. For each tested material, 40 cylindrical specimens (8-mm diameter, 4-mm height) were prepared. Specimens were divided into 4 subgroups according to polishing system used (*n* = 10/subgroup): subgroup I; Mylar strip (control), subgroup II; 1-step polishing system, subgroup III; 2-step polishing system, and subgroup IV; multi-step polishing system. Surface roughness was evaluated with a 3D noncontact optical profilometer. Gloss was evaluated with a glossmeter. Microhardness was evaluated with a Vickers Microhardness tester. Statistical analysis was performed using two-way ANOVA, Bonferroni, and Pearson correlation coefficient testes (*p* < 0.05).

**Results:**

For surface roughness, there was no significant difference between groups for control and 1-step (*p* = 0.152 for control, *p* = 0.296 for 1-step), while there was a significant difference for 2-step and multi-step (*p* = 0.025 for 2-step, *p* = 0.001 for multi-step). For gloss, there was no significant difference between groups for 1-step and 2-step (*p* = 0.124 for 1-step, *p* = 0.226 for 2-step), while there was a significant difference for control and multi-step (*p* = 0.001 for control, *p* < 0.001 for multi-step). For microhardness, there was no significant difference between groups for control (*p* = 0.245), while there was a significant difference for the other techniques (*p* < 0.001 for 1-step and 2-step, *p* = 0.001 for multi-step).

**Conclusions:**

Reinforcement with zirconium oxide fillers improved flowable bulk-fill composites in terms of surface roughness, gloss, and microhardness. Surface gloss and microhardness were significantly influenced by material type and polishing system used, while surface roughness was influenced only by polishing system used.

## Introduction

Dental resin composites are becoming the most popular direct restorative material for both anterior and posterior teeth due to patients’ increasing esthetic demands [1, 2]. Additionally, dental resin composites are now the preferred restorative material in most nations due to the detrimental environmental consequences of dental amalgam [3]. Dental resin composites are primarily composed of a matrix that is responsible for the polymerization reaction, and filler particles that are mixed into the resin matrix to improve the composites’ mechanical and physical characteristics. Fillers also reduce the polymerization shrinkage which is the main shortage of the resin matrix [4]. Several advancements have been made to improve the mechanical and physical characteristics by modifying the matrix and fillers to extend the lifespan and therapeutic use of resin composite materials [5].

Fillers have passed through several phases of development in various aspects until nanotechnology was used [6]. These nanofillers may consist of nanoparticles or nanoaggregates made of high filler load nanoparticles of silica or zirconium/silica [7]. Resin composites’ mechanical and physical characteristics were enhanced, and their polymerization shrinkage was reduced [8].

The dental industry has seen the introduction of novel “bulk-fill” resin composites, which have the ability to polymerize into thicknesses of up to 4 and 5 mm [9, 10]. Different initiators, filler technologies, translucent structures, and alternate organic matrices are the basic characteristics of these resin composites. Therefore, bulk-fill resin composites may present advantages such as time savings and easier clinical applications [11, 12]. Bulk-fill composite resins may reduce shrinkage stress and simplify clinical processes, so they have become more popular [13]. More compact restorations may be created since the bulk-fill placement prevents contamination and gaps from forming between composite layers. Increased light transmittance through bulk-fill resin composite, which is achieved by enhancing its transparency, can result in more successful restorations. Bulk-fill resin composites can be divided into two categories according to consistency: regular viscosity and low viscosity materials [14].

Flowable bulk-fill resin composites (FBRCs) are becoming more and more common because they are simpler to manipulate with than regular viscous formulations and have excellent flow qualities that enable both the penetration of very narrow, deep clefts and the rapid filling of large gaps. The size and quantity of filler determine reduced viscosity and customized flowability; as a result, flowable bulk-fill resin composites are continually provided with less filler, which adversely affects a number of mechanical characteristics, particularly the elastic modulus and wear resistance [15, 16]. Flowable bulk-fill resin composites were believed they need to be capped with a layer of regular or high viscosity resin composite as they had inadequate color stability and low wear resistance [17]. Recently, with more advancement of fillers and resin matrix, FBRCs can be adapted to irregular anterior and posterior cavities and don’t require a capping layer of regular viscosity resin composite. Also, increasing filler load and using spherical nano zirconia and silica fillers provide good polishability and enhance the physical, mechanical, and esthetic properties. Besides that, their flowable nature provides excellent cavity adaptation and more time-saving procedures [18, 19].

Surface characteristics affect the durability and clinical behavior of restorative materials [20]. Rough surfaces make restorations more susceptible to the accumulation of plaque, gingival irritation, discoloration, and recurrent caries, so surface roughness has a significant impact on the esthetics and durability of restorations [21–24]. Similarly, the human eye can detect changes in gloss, so it is essential to ensure matching between the restoration and the surrounding enamel [25]. Microhardness is a crucial surface characteristic that establishes the material’s ability to withstand long-term scratches [26, 27]. Since resin composite with higher microhardness has more wear resistance [28, 29]. The dental resin composite composition and the restoration’s finishing or polishing method affect the surface roughness, gloss, and microhardness [30–32].

Resin composite finishing and polishing (F/P) are crucial procedures during restoration [33, 34]. Despite a properly placed Mylar strip resulting in the cleanest and most polished surface possible, the cured composite surface beneath it is rich in resin and may include voids and defects [35, 36]. Additionally, resin composite restorations require shape correction, flash removal, and modification [37]. The surface is rough after finishing processes and requires further polishing [38, 39]. The endurance and esthetics of resin composite restorations are impacted by finishing and polishing [40, 41]. Achieving desirable anatomy and appropriate occlusion is provided by proper F/P. Additionally, proper F/P lessens SR, scratches, and their effects and provides a smooth, hard, glossy and esthetically stable surface finish [42]. The dental market offers a wide range of F/P techniques and systems with varying compositions and steps. Different types of F/P techniques, such as coated abrasive discs (finishing discs), carbide burs, abrasive stones, diamond burs, abrasive-impregnated rubber wheels and cups, abrasive strips, and polishing pastes, differ in their effects [43–45]. The multi-step systems use a smaller grain polish system at each step to remove scratches in the previous polisher until a high gloss surface is obtained. For single-step systems, abrasive size is important due to the possibility of leaving scratches on the resin composites. Therefore different F/P systems with different steps should be evaluated with different types of resin composites [46].

It was reported that, the hardness of fillers, as long as the different F/P systems were found to have a significant effect on SR, SG and microhardness [47, 48]. Adding radiopaque zirconium oxide (ZrO_2_) nanofillers, which are harder and have higher wear resistance than conventional fillers, to FBRCs would enhance mechanical properties and help to improve radiographic diagnosis [49–52].

A comprehensive search of the literature revealed that there are no published studies assessing the effects of various finishing and polishing techniques on FBRCs reinforced with ZrO_2_ fillers and how these procedures affect the surface roughness, gloss, and microhardness of these resin composite materials according to the knowledge of authors. Therefore, more researches were required to expand and strengthen the scientific information regarding this issue. This study was designed to evaluate the impact of different polishing systems on surface roughness, surface gloss, and vickers microhardness of flowable bulk-fill composites reinforced with zirconium oxide fillers. The first null hypothesis tested was that the type of flowable bulk-fill resin composite had no significant effect on surface roughness, surface gloss, and microhardness. The second null hypothesis tested was that finishing and polishing technique had no significant effect on surface roughness, surface gloss, and microhardness of all tested flowable bulk-fill composite. The third null hypothesis tested was that there was no correlation between surface roughness, surface gloss, and microhardness regarding the type of resin composite and the type of finishing and polishing system.

## Materials and methods

### Materials

In this study, the resin composite materials used were: two commercially available FBRCs reinforced with silica-zirconia fillers; PALFIQUE BULK FLOW (PBF) (Tokuyama Dental, Tokyo, Japan), and Filtek Bulk Fill Flowable (FBF) (3 M ESPE St. Paul, MN, USA), and another FBRC with conventional fillers; Tetric N-Flow Bulk Fill (TBF) (Ivoclar Vivadent AG, Schaan, Liechtenstein). Materials used in this study are shown in Table 1. The F/P systems used in this study were: a 1-step F/P system; OneGloss (Shofu, Japan), a 2-step F/P system; Sof-Lex Spiral (3 M ESPE St. Paul, MN, USA), and a multi-step F/P system; OptiDisc (Kerr, USA). F/P systems and techniques are shown in Table 2.

**Table 1:** Materials used in the study

Material	Composition	Manufacturer	Lot No.
PALFIQUE BULK FLOW	Matrix: 2-hydroxy propoxy, Bis-GMA, Bis-MPEPP, TEGDMA, Mequinol, Dibutyl hydroxyl toluene, and UV absorber Fillers: Supra-Nano Spherical filler (200 nm spherical SiO2-ZrO2) Filler loading: 70 wt% (56 vol%)	Tokuyama Dental, Tokyo, Japan	131E22
Filtek Bulk Fill Flowable	Matrix: Bis-GMA, TEGDMA, Bis-EMA, and procrylat resins Fillers: ytterbium, trifluoride (size range from 0.1 to 5.0 microns) and SiO2-ZrO2 (size range from 0.01 to 3.5 microns) Fillers loading: 64.5 wt% (42.5 vol%)	3 M ESPE (St. Paul, MN, USA)	10,073,908
Tetric N-Flow Bulk Fill	Matrix: monomethacrylates and dimethacrylates Fillers: barium glass, ytterbium trifluoride and copolymers (ranges between 0.1 μm and 30 μm with a mean particle size of 5 μm) Fillers loading: 68.2 wt% (46.4 vol%)	Ivoclar Vivadent AG, Schaan, Liechtenstein	Z0417F

**Table 2:** Finishing and Polishing systems used in the study

	Material & Lot No.	Manufacturer	Procedures
1-step	OneGloss Lot No: 0721327	Shofu, Japan	Used for 30 s with a low-speed handpiece for each specimen at a rotation speed 10,000 RPM with intermittent water spray heavy pressure was applied first for finishing then light pressure for polishing.
2-step	Sof-Lex Spiral Finishing and Polishing Wheels Lot No: N995293	3 M ESPE (St. Paul, MN, USA)	Pre-polishing with rubber spiral coated with aluminium oxide (beige) for 30 s, followed by diamond polishing spiral wheel (pink) for 30 s with a low speed handpiece at 15,000 RPM with intermittent water spray.
Multi-step	OptiDisc Lot No: 9,589,808	Kerr, USA	descending sequence of abrasiveness, medium (light brown), fine (orange), and superfine (yellow) with uniform pressure and a planar motion using low speed handpiece at 10,000 RPM for 15–20 s for each disc with intermittent water spray

### Methods

#### Sample size calculation and study design

The sample size was calculated based on surface roughness results retrieved from a previous study [53]. Based on the effect size of 1.05, a 2-tailed test, α error = 0.05, and power = 90.0%, the sample size was determined to be 9 in each group using the G power software version 3.1.9.7. Therefore, 10 specimens were included in each group. Three groups of specimens were created based on the type of FBRC (*n* = 40). Each group was divided into four subgroups based on the type of F/P technique used (*n* = 10/subgroup). Subgroup I: a control group (Mylar strip) where no finishing technique was applied, subgroup II: specimens were finished with 1-step technique, subgroup III: specimens were finished with 2-step technique, and subgroup IV: specimens were finished with multi-step technique. The finishing and polishing procedures were done by the same operator to avoid bias, using a low-speed contra-angle handpiece (Sirona, Dentsply, USA) with water coolant. Specimens were fixed in a base made of dental stone and a spirit level was used to make sure they were parallel to the floor. New discs and polishers were used to polish each specimen. Surface roughness, surface gloss, and Vickers microhardness tests were performed on the same specimens.

#### Specimens’ preparation

All specimens were prepared using split cylindrical Teflon molds (8-mm diameter and 4-mm height). A Mylar strip was applied over a glass slide, and then the Teflon mold was fixed on them. Composite resin materials were injected into the mold until the mold was slightly overfilled, and then the mold was covered by another Mylar strip and glass slide. The glass slide above the mold was pressed with a constant metal weight (500 g) for 30 s to eliminate any excess material and make the surface flat. A light-emitting diode (LED) curing device (Elipar S10, 3 M ESPE) calibrated at 1,200 mW/cm^2^ for 20 s was then used to polymerize the specimens. Irradiance of the light curing unit was regularly checked by a radiometer (Bluephase Meter II, Ivoclar Vivadent, Liechtenstein). After the specimens were removed from the mold, each specimen has been marked from its side to distinguish the upper surface from the lower surface. Then all specimens were kept in distilled water at 37 ± 1 °C for 24 h before F/P procedures. F/P systems used and the technique used for each system are presented in Table 2 in details. After polishing the upper surfaces of all specimens, the specimens were kept in distilled water at 37 ± 1 °C for 24 h before evaluating the surface roughness, gloss, and microhardness of the polished surfaces.

#### Surface roughness evaluation

**Fig. 1:**
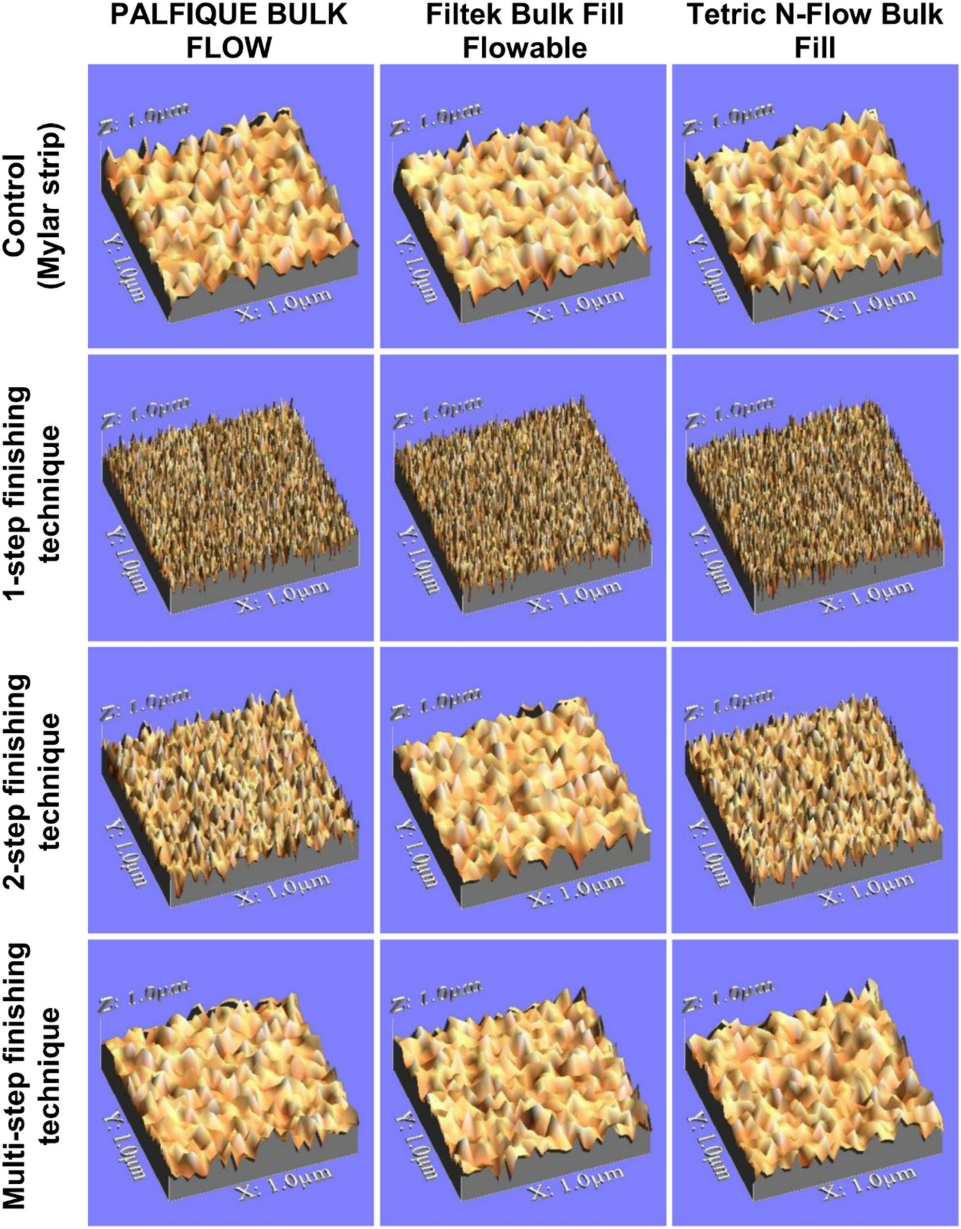
Surface topography of representative specimens from all tested groups. Specimens were photographed using a USB Digital microscope attached to a built-in camera (Scope Capture Digital Microscope, Guangdong, China) connected to IBM compatible computer at a magnification of 120X with a resolution of 350 × 400 pixels. A 3D image was created using a digital image analysis system WSxM software (Ver 5 develop 4.1, Nanotec, Electronica, SL)

A 3D noncontact optical profilometer (Model NT 1100, Wyko, Veeco, USA) coupled to a PC running image software (Vision 32, Veeco, USA) was used to measure SR [54, 55]. The program presents the images that gave arithmetic roughness mean (Ra) data based on the peaks and valleys observed in the analyzed region using a profilometer with a 2.4 mm evaluation length and a 0.8 mm cut-off. Consequently, a three-dimensional image of the specimen’s surface was produced. After that, five 3D pictures of each specimen were taken, one in the middle and one on each side [56]. Surface topography of representative specimens from all tested groups is shown in Fig. 1.

#### Gloss evaluation

A ZHENTNER glossmeter of type ZGM 1130 (supplied by ZHENTNER testing equipment– Switzerland) was used to measure the gloss of every specimen. A black glass standard has a gloss of 100 gloss units (GU), which considered the perfect gloss and GU of 0 considered nonglossy, was used as a reference [57]. The measurement area was 2 × 2 mm and 60º geometry angel was used to assess each specimen [58, 59]. Each specimen was placed underneath the device base and eventually covered the whole measuring area in the center of the aperture to collect the measurements; each specimen had three measurements, which were then averaged. To avoid any outside light during the measurement process, the measurements were conducted in a completely dark room.

#### Microhardness evaluation

The specimens’ surface microhardness was measured using the Digital Display VMH Tester (Model HVS-50, Laizhou Huayin Testing Instrument, China) with a 20X objective lens and a diamond Vickers indenter. A 100 g force was applied to the specimens’ surface for 15 s. Each specimen’s surface had three indentations made at least 0.5 mm apart. Following the measurement of the diagonal length of the indentations using an integrated scaled microscope, Vickers values were converted into microhardness values. An equation was then used to calculate the microhardness: HV = 1.854 P/d^2^, where HV is Vickers hardness in Kgf/mm^2^, P is the load in Kgf and d is the length of the diagonals in mm.

### Statistical analysis

The data were analyzed using SPSS^®^ software version 25 (SPSS Inc., Chicago, IL, USA). Shapiro-Wilk test was used to diagnose the normality of data distribution of all variables. The data were parametric and met the normal distribution. Consequently, descriptive statistics were presented using mean, and standard deviation (SD). Comparisons for surface roughness, surface gloss, and microhardness between groups (Three different FBRCs) and F/P techniques (control, 1-step, 2-step, and multi-step) were made using two-way ANOVA followed by Bonferroni test for multiple comparisons. A correlation between the tested parameters was measured using the Pearson correlation coefficient. *p* < 0.05 was considered to be significant.

## Results

### Surface roughness test results

Two-way ANOVA test showed no significant difference in overall surface roughness between FBRCs (*p* = 0.147). However, there was a significant difference in overall surface roughness between F/P techniques (*p* < 0.001). Also, the interaction FBRC*F/P technique was significant (*p* < 0.001).

**Fig. 2:**
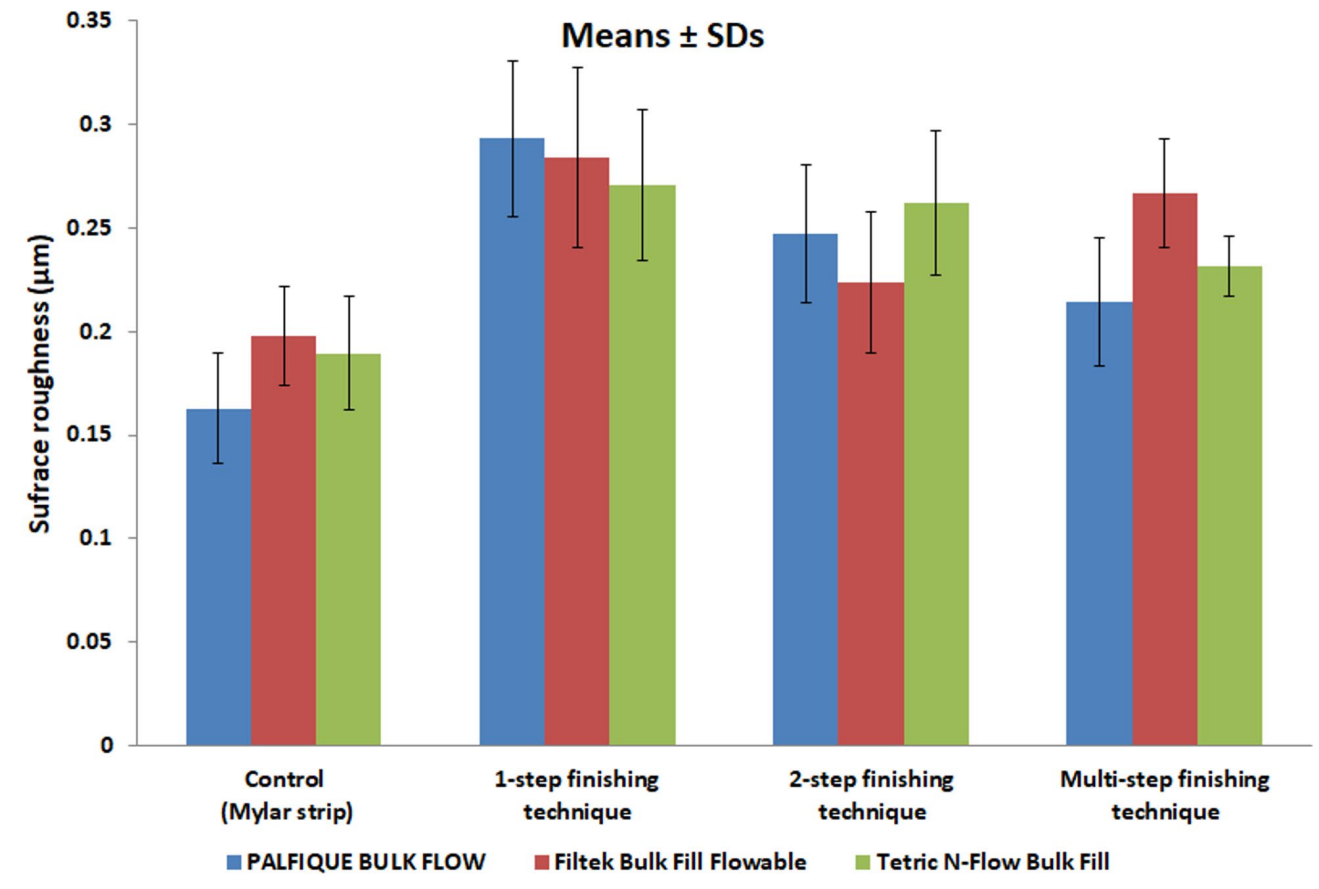
A bar chart showing mean values and SDs of surface roughness (in µm) of all tested flowable bulk-fill resin composites for each finishing and polishing technique

Comparison of surface roughness between FBRCs for each finishing and polishing technique is presented in Table 3; Fig. 2. For control and 1-step, there was no significant difference in SR among tested FBRCs (*p* = 0.152 for control, and *p* = 0.296 for 1-step). For the 2-step and multi-step, there was a significant difference in SR between tested FBRCs (*p* = 0.025 for 2-step, and *p* = 0.001 for multi-step). For 2-step, Tetric N-Flow Bulk Fill had the highest SR, PALFIQUE BULK FLOW came in second, and Filtek Bulk Fill Flowable had the lowest SR. For multi-step, Filtek Bulk Fill Flowable had the highest SR, Tetric N-Flow Bulk Fill came in second, and PALFIQUE BULK FLOW had the lowest SR. Multiple (post hoc) comparisons between each tested FBRCs are showed in the same table. For 2-step, and multi-step finishing techniques, there was a significant difference between PALFIQUE BULK FLOW and Filtek Bulk Fill Flowable, as well as between Filtek Bulk Fill Flowable and Tetric N-Flow Bulk Fill. However, PALFIQUE BULK FLOW and Tetric N-Flow Bulk Fill did not differ significantly.

**Table 3:** Comparison between surface roughness mean values (SDs) in micrometer (µm) among all tested flowable bulk-fill resin composites and finishing techniques used

	Control	1-step	2- step	Multi-step	Two-way ANOVA (*p* value)
PALFIQUE BULK FLOW	0.16 (0.03) ^Aa^	0.30 (0.04) ^Ba^	0.25 (0.03) ^Ca^	0.21 (0.03) ^Da^	< 0.001*
Filtek Bulk Fill Flowable	0.20 (0.02) ^Aa^	0.29 (0.04) ^Ba^	0.22 (0.03) ^Cb^	0.27 (0.03) ^Bb^	< 0.001*
Tetric N-Flow Bulk Fill	0.19 (0.03) ^Aa^	0.27 (0.04) ^Ba^	0.26 (0.04) ^Ba^	0.23 (0.01) ^Ca^	< 0.001*
Two-way ANOVA (*p* value)	0.152	0.296	0.025*	0.001*	

^A, B^ Different superscripted capital letters in same row indicate statistically significant differences

^a, b^ Different superscripted lowercase letters in same column indicate statistically significant differences

Comparison of surface roughness between types of F/P technique for each tested FBRC is presented in Table 3. For all tested FBRCs, there was a significant difference in SR between F/P techniques (*p* < 0.001 for all tested FBRCs). For PALFIQUE BULK FLOW and Tetric N-Flow Bulk Fill, the highest SR was observed with 1-step, followed by 2-step, then multi-step, and the lowest SR was noted with the control. For Filtek Bulk Fill Flowable, the highest SR was observed with 1-step, followed by multi-step, then 2-step, and the lowest SR was noted with control. Multiple (post hoc) comparisons between each two F/P techniques are shown in the same table. For all tested FBRCs, there was a significant difference between each two finishing techniques, except between 1-step and multi-step for Filtek Bulk Fill Flowable, and between 1-step and 2-step for Tetric N-Flow Bulk Fill.

### Surface gloss test results

Two-way ANOVA test showed that there was a significant difference in overall surface gloss between FBRCs (*p* < 0.001) and F/P techniques (*p* < 0.001). Also, the interaction FBRC*F/P technique was significant (*p* < 0.001).

**Fig. 3:**
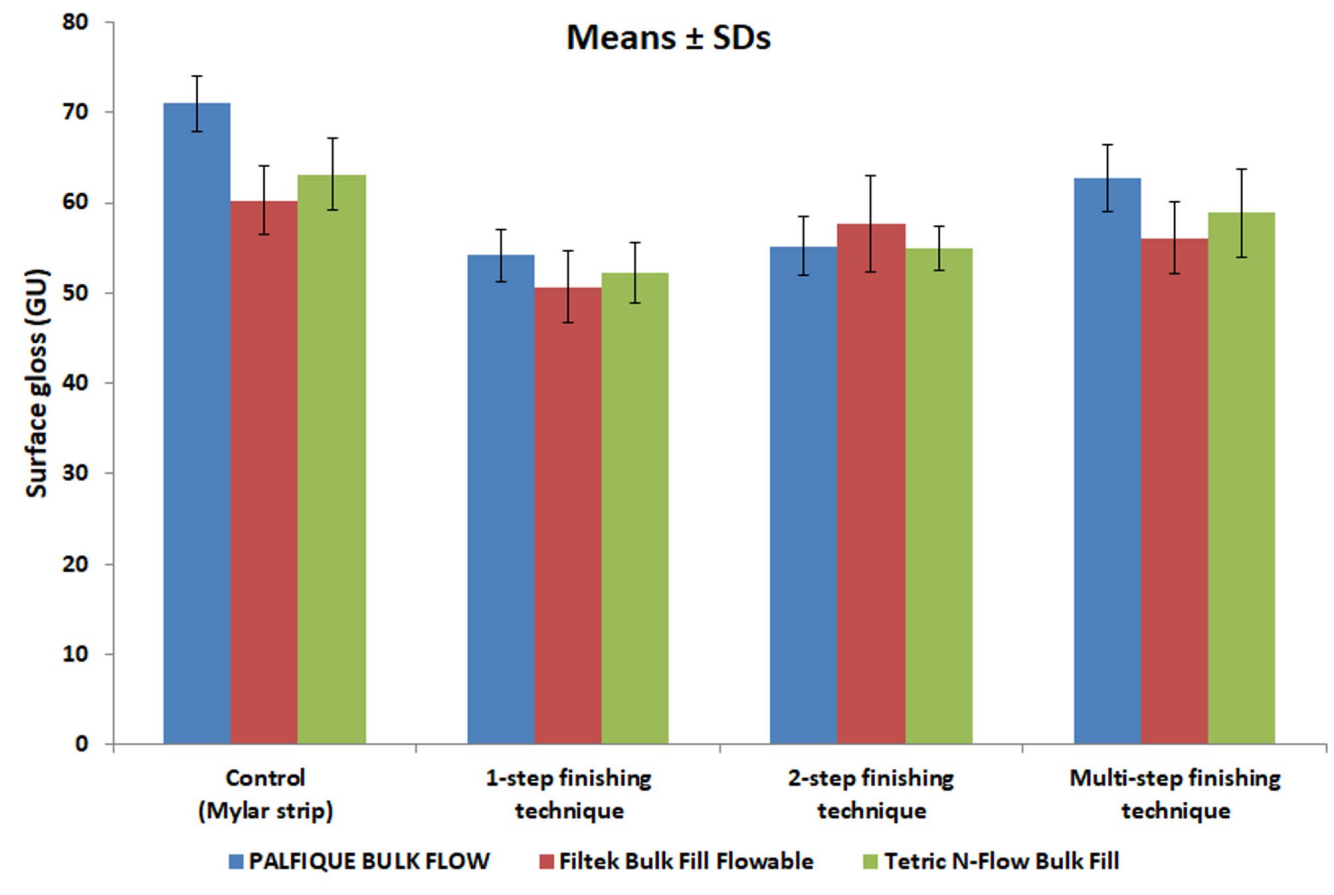
A bar chart showing mean values and SDs of surface gloss (in GU) of all tested flowable bulk-fill resin composites for each finishing and polishing technique

Comparison of surface gloss between FBRCs for each F/P technique is presented in Table 4; Fig. 3. For 1-step and 2-step, there was no significant difference in SG between tested FBRCs (*p* = 0.124 for 1-step, and *p* = 0.226 for 2-step). For control and multi-step, there was a significant difference in SG between FBRCs (*p* = 0.001 for control and *p* < 0.001 for multi-step). For control, 1-step, and multi-step, PALFIQUE BULK FLOW produced the highest SG, Tetric N-Flow Bulk Fill came in second, and Filtek Bulk Fill Flowable had the lowest surface gloss. For 2-step, Filtek Bulk Fill Flowable had the greatest SG, PALFIQUE BULK FLOW came next, and Tetric N-Flow Bulk Fill showed the lowest surface gloss. Multiple (post hoc) comparisons between each two FBRCs is presented in the same table. For control and multi-step F/P technique, there was a significant difference between PALFIQUE BULK FLOW and Filtek Bulk Fill Flowable, as well as between PALFIQUE BULK FLOW and Tetric N-Flow Bulk Fill. However, Filtek Bulk Fill Flowable and Tetric N-Flow Bulk Fill did not differ significantly.

**Table 4:** Comparison between surface gloss mean values (SDs) in gloss units (GU) among all tested flowable bulk-fill resin composites and finishing techniques used

	Control	1-step	2-step	Multi-step	Two-way ANOVA (*p* value)
PALFIQUE BULK FLOW	70.96 (3.12) ^Aa^	54.17 (2.88) ^Ba^	55.21 (3.21)^Ba^	62.72 (3.73) ^Ca^	< 0.001*
Filtek Bulk Fill Flowable	60.26 (3.87) ^Ab^	50.67 (3.92) ^Ba^	57.63 (5.30) ^ACa^	56.07 (3.99) ^Cb^	< 0.001*
Tetric N-Flow Bulk Fill	63.13 (4.01) ^Ab^	52.27 (3.36) ^Ba^	54.96 (2.44) ^Ba^	58.84 (4.82) ^Cb^	< 0.001*
Two-way ANOVA (*p* value)	0.001*	0.124	0.226	< 0.001*	

^A, B^ Different superscripted capital letters in same row indicate statistically significant differences

^a, b^ Different superscripted lowercase letters in same column indicate statistically significant differences

Comparison of surface gloss between types of F/P technique for each FBRC is presented in Table 4. For all tested FBRCs, there was a significant difference in SG between F/P techniques (*p* < 0.001 for all groups). For PALFIQUE BULK FLOW and Tetric N-Flow Bulk Fill groups, the highest SG was observed with control, followed by multi-step, then 2-step, and the lowest surface gloss was noted with 1-step. For Filtek Bulk Fill Flowable, the highest SG was observed with control, followed by 2-step, then multi-step, and the lowest SG was noted with 1-step. Multiple (post hoc) comparisons between each two F/P techniques are presented in the same table. For PALFIQUE BULK FLOW and Tetric N-Flow Bulk Fill groups, there was a significant difference between each two F/P techniques except between the 1-step and 2-step. For Filtek Bulk Fill Flowable group, there was a significant difference between each two F/P techniques except between control and 2-step, and between 2-step and multi-step.

### Vickers microhardness test results

Two-way ANOVA test showed that there was a significant difference in overall VMH between FBRCs (*p* < 0.001) and F/P techniques (*p* < 0.001). However, the interaction FBRC*F/P technique was not significant (*p* = 0.365).

**Fig. 4:**
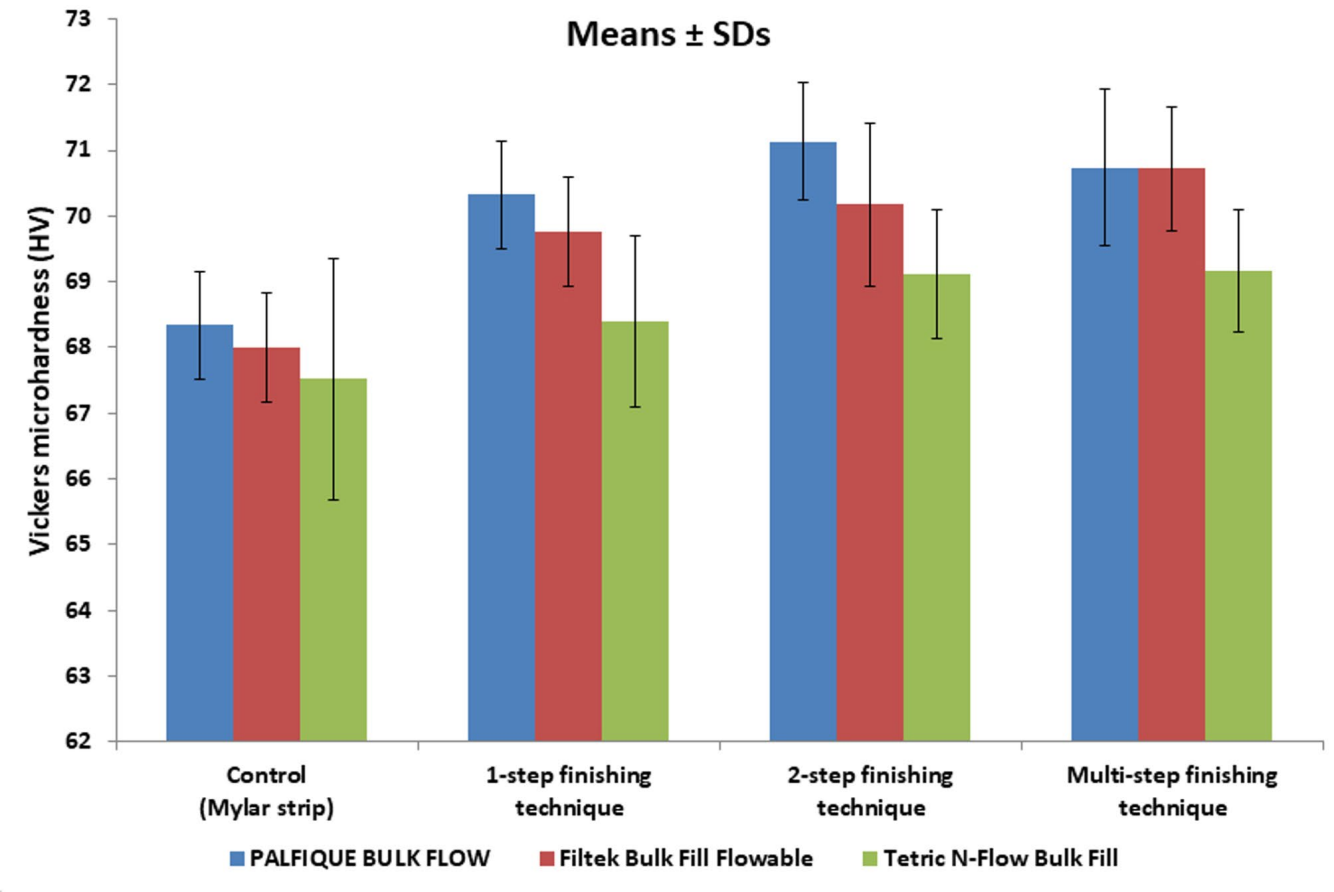
A bar chart showing mean values and SDs of Vickers microhardness (in HV) of all tested flowable bulk-fill resin composites for each finishing and polishing technique

Comparison of VMH between FBRCs for each F/P technique is presented in Table 5; Fig. 4. For control, there was no significant difference in VMH between FBRCs (*p* = 0.245). For the other F/P techniques, there was a significant difference in VMH between FBRCs (*p* < 0.001 for 1-step and 2-step, and *p* = 0.001 for multi-step). For all F/P techniques, PALFIQUE BULK FLOW had the highest VMH, followed by Filtek Bulk Fill Flowable, while Tetric N-Flow Bulk Fill showed the lowest microhardness. Multiple (post hoc) comparisons between each two FBRCs are showed in the same table. For 1-step, 2-step, and multi-step F/P techniques, there was a significant difference between PALFIQUE BULK FLOW and Tetric N-Flow Bulk Fill, as well as between Filtek Bulk Fill Flowable and Tetric N-Flow Bulk Fill. However, PALFIQUE BULK FLOW and Filtek Bulk Fill Flowable did not differ significantly.

**Table 5:** Comparison between microhardness mean values (SDs) in Vickers hardness number (HV) among all tested flowable bulk-fill resin composites and finishing techniques used

	Control	1-step	2-step	Multi-step	Two-way ANOVA (*p* value)
PALFIQUE BULK FLOW	68.34 (0.82) ^Aa^	70.32 (0.81) ^Ba^	71.13 (0.89) ^Ba^	70.74 (1.20) ^Ba^	< 0.001*
Filtek Bulk Fill Flowable	68.00 (0.84) ^Aa^	69.76 (0.84) ^Ba^	70.17 (1.25) ^Ba^	70.72 (0.95) ^Ba^	< 0.001*
Tetric N-Flow Bulk Fill	67.52 (1.84) ^Aa^	68.40 (1.3) ^Bb^	69.11 (0.98) ^Bb^	69.16 (0.93) ^Bb^	0.005*
Two-way ANOVA (*p* value)	0.245	< 0.001*	< 0.001*	0.001*	

^A, B^ Different superscripted capital letters in same row indicate statistically significant differences

^a, b^ Different superscripted lowercase letters in same column indicate statistically significant differences

Comparison of VMH between types of F/P technique for each FBRC is presented in Table 5. For all tested FBRCs, there was a significant difference in VMH between finishing techniques (*p* < 0.001 for PALFIQUE BULK FLOW and Filtek Bulk Fill Flowable, and *p* = 0.005 for Tetric N-Flow Bulk Fill). For Filtek Bulk Fill Flowable and Tetric N-Flow Bulk Fill, the highest VMH was observed with the multi-step, followed by the 2-step, then the 1-step, and the lowest VMH was noted with control. For PALFIQUE BULK FLOW, the highest VMH was observed with the 2-step, followed by the multi-step, then the 1-step, and the lowest VMH was noted with control. Multiple (post hoc) comparisons between each two F/P techniques are presented in the same table. For all FBRCs, there was a significant difference between control and other F/P techniques (1-step, 2-step, and multi-step). However, there was no significant difference between 1-step, 2-step, and multi-step F/P techniques.

### Correlation between parameters

**Fig. 5:**
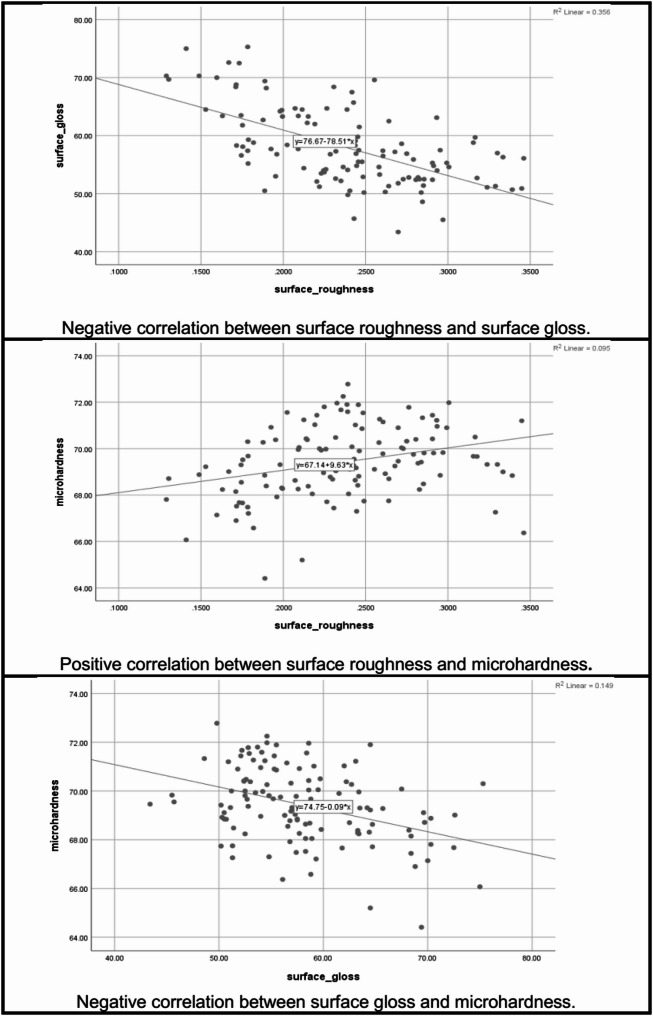
Linear charts showing the correlations between surface roughness, surface gloss and microhardness for all tested flowable bulk-fill resin composites

Correlation between tested parameters is presented in Table 6; Fig. 5. There was a significant negative correlation between surface roughness and surface gloss (*R*=-0.597, *p* < 0.001). There was a significant positive correlation between surface roughness and microhardness (*R* = 0.307, *p* < 0.001). There was a significant negative correlation between surface gloss and microhardness (*R*=-0.385, *p* < 0.001).

**Table 6:** Correlations between all tested parameters

		Correlations		
		Surface roughness	Surface gloss	Microhardness
Surface roughness	Pearson Correlation	1	-0.597^**^	0.307^**^
	Sig. (2-tailed)		0.000	0.001
	N	120	120	120
Surface gloss	Pearson Correlation	-0.597^**^	1	-0.385^**^
	Sig. (2-tailed)	0.000		0.000
	N	120	120	120
Microhardness	Pearson Correlation	0.307^**^	-0.385^**^	1
	Sig. (2-tailed)	0.001	0.000	
	N	120	120	120

**. Correlation is significant at the 0.01 level (2-tailed)

## Discussion

Over the years, the development of resin composite has continuously advanced [5]. Nowadays, fillers used in resin composites typically consist of several components, mainly particulate, silica-based fillers, with alumina, zirconia, or any other metal oxides, either separately or in combination, to achieve acceptable mechanical properties, color, and transparency [7]. Furthermore, it has been established that zirconia significantly strengthens the mechanical characteristics of resin composites [50, 51]. Additionally, manufacturers have introduced FBRCs, which have improved curing, shrinkage, mechanical, and wear qualities [14]. This study assessed the surface roughness, surface gloss, and surface microhardness of FBRCs reinforced with zirconium oxide fillers (PALFIQUE BULK FLOW and Filtek Bulk Fill Flowable) in comparison to another bulk-fill flowable resin composite with conventional fillers (Tetric N-Flow Bulk Fill).

The effectiveness of polishing techniques is based on several variables, including the flexibility of the backing material in which the abrasive particles are embedded, the hardness of the abrasives themselves, the geometry of the polishing tools, and the application method (i.e., pressure, duration, and angle). When polishing composite materials, the particles in the abrasive must be harder than the filler particles in the resin. If not, polishing will only occur by the softer resin matrix being worn away, with the harder fillers left behind as protrusions and producing a rough, uneven surface [45]. Since most studies have shown that discs containing aluminium oxide particles are an effective tool for creating a smooth surface [2, 23], OptiDiscs had been used in the current study. Spiral polishing wheels were chosen for our study because of their design, since they can polish any flat or curved surface, including the occlusal surface of posterior teeth [9, 58]. The polishing systems in this study were chosen depending on the number of application steps: 1-step F/P system (OneGloss), 2-step F/P system (Sof-Lex Spiral), and multi-step F/P system (OptiDisc).

In this study, a single operator handled every attempt to guarantee that the technique and all of processes were standardized. To create a uniform, smooth surface for every specimen, the resin composite material was pressed by a constant metal weight between two thin glass slides that were coated on both sides with Mylar strips. Procedures for F/P were delayed for 24 h in this study. Since some earlier research showed that immediate F/P might result in insufficient maturation and plastic deformation of the resin, due to being prone to heat generation [34, 42]. Nasoohi et al. [36] found that dry finishing and polishing resin composites can increase SR values because the abrasive particles detached from the polishing tool may become lodged in the surface of resin composite. On the other hand, Kaminedi et al. [24] found that the smoothest and highest hardness values in nanohybrid resin composites were obtained with dry finishing and polishing. Despite the debate over whether the approach produces smoother surfaces, the generation of excessive heat that might arise from dry F/P and weaken the matrix/filler connection must be taken into account. In the current study, all finishing and polishing systems were used according to manufacturer’s instructions under intermittent water spray.

The surface’s roughness is one of numerous indicators used to describe how a surface deviates from its ideal condition due to slight imperfections that alter the surface’s smoothness, which is impacted by the materials or the manufacturing process [1, 20]. Because a non-contact digital profilometer microscope can scan a surface using a certain kind of laser and produce a 3D surface map without harming the specimens, it was chosen in this study as a quick and simple assessment tool [54, 60]. Surface roughness above the roughness threshold (Ra = 0.2 μm) simultaneously increases biofilm buildup, and below the threshold value, no further bacterial adherence is detectable [55]. Smooth surfaces increase patient comfort since the tip of the tongue can detect a 0.3 μm difference in SR [37]. In the current study, all F/P systems produce surface roughness less than 0.3 μm. So all three systems used in this study could be accepted clinically.

Gloss, which is measured in GU, is a visual appearance characteristic that includes specular reflection from a surface [58]. Numerous factors influence gloss, such as filler size, filler-matrix uniformity, and the refractive index difference between the resin matrix and the fillers [58, 59]. High-gloss resin composite materials blend in better with adjacent teeth than low-gloss ones, and gloss values correlate with resin composite smoothness [59]. Da Costa et al. [57] said that gloss levels between 40 and 50 GU are clinically adequate for resin composites; however previous studies indicated that gloss values between 60 and 70 GU are acceptable [9, 29, 58].

Microhardness is a feature of resin composite that affects its endurance and is linked to its wear resistance [31]. By using the right polishing techniques to remove the outer layer, restoration surfaces with high hardness and wear resistance are produced [22]. The amount of applied force divided by the exposed contact area is the way we can measure the microhardness. The Vickers and Knoop tests are the primary methods used to measure it. Their indenters are shaped differently. In contrast to the diamond base in Knoop, the indenter in the Vickers microhardness testing has a square base pyramid form. In this study, surface microhardness was assessed using the Vickers tester. Because the diagonal indentations that were created by the indenter should be sufficiently large to allow for maximum resolution for accurate measurement, a 100 g applied force was used. If the load exceeded this, the indentation could be too big for the screen [39]. Additionally, the measurements may be impacted by cracks in the indentation.

In this study, surface roughness was significantly influenced by the polishing systems but less so by resin composite type. Babina et al. [33] stated that the resin composite type has an insignificant impact on the restoration SR. In contrast, other studies found that SR was significantly impacted by polishing systems, along with the type of resin composite [10, 11]. In this study, the control group, where samples were cured against a Mylar strip, produced the lowest surface roughness values, which agrees with previous studies [21, 32, 35]. A relatively higher level of surface roughness results from the hard filler particles that are abraded away from the resin matrix when the surface of resin composite comes into contact with finishing and polishing techniques [21]. Among F/P systems, the multi-step F/P system resulted in the lowest SR, followed by 2-step and 1-step, respectively. This could be related to the uniform removal of resin matrix and fillers from the surface by polishing systems that use aluminium oxide-impregnated discs, as previous studies found that flexible aluminium oxide discs resulted in minimum surface roughness in resin composite [10, 34, 45]. According to Erturk-Avunduk et al. [21] and Batmaz et al. [41] multi-step polishing technique tend to have better SR than 1-step technique which agrees with our study. In contrast, Korkmaz et al. [38] found that the 1-step polishing technique results in SR comparable to the multi-step polishing system, and that could be because of the different multi-step system used. Among resin composite materials, PBF exhibited the smoothest surface with the multi-step F/P technique. For the 2-step F/P technique, FBF demonstrated the lowest roughness values. This means, ZrO_2_-containing resin composite exhibited the smoothest surface, which agrees with previous studies found that resin composites reinforced with ZrO_2_ fillers exhibited lower surface roughness [48, 49].

In the current study, surface gloss showed significant difference with resin composite type and finishing techniques, which agrees with previous studies [2, 35]. Among the materials, PBF exhibited the highest gloss for most F/P techniques, followed by TBF and FBF respectively. This hierarchy slightly varied with the 2-step F/P technique, as FBF achieved the highest gloss. The multi-step polishing system produced the highest gloss values across most groups, followed by the 2-step system and the 1-step system. Pala et al. [29] and Altınışık et al. [32] reported that 1-step polishing technique (OneGloss) resulted in the least gloss among the other polishing techniques. Also Rodrigues-Junior et al. [25] and Nithya et al. [53] reported that multi-step F/P systems produced higher SG than produced by 1-step systems. The control group (Mylar strip) generally exhibited highest SG values for resin composites due to the smooth surface imparted by Mylar strips during specimens preparation, as confirmed by previous studies [26, 35].

The results demonstrated significant differences in microhardness across FBRC types and finishing techniques, and this revealed that both the type of FBRC and finishing technique significantly influenced VMH. Alharbi et al. [44] and Ehrmann et al. [39] agreed with this. Among the tested materials, the resin composites reinforced with ZrO_2_ fillers exhibited higher VMH compared to resin composites with conventional fillers. PBF exhibited the highest microhardness, TBF exhibited the lowest microhardness, and FBF exhibited intermediate microhardness. Previous studies found that the resin composite with higher filler load resulted in increased microhardness [6, 27]. In this study, this might explain that PBF (70 wt% and 56 vol%) had a higher VMH than TBF (filler load 68.2 wt% and 46.4 vol%) and FBF (filler load 64.5 wt% and 42.5 vol%). Although TBF has higher filler load than FBF, VMH of FBF was higher than VMH of TBF. The presence of ZrO_2_ fillers in FBF might explain this result. The enhanced microhardness observed in this study with all F/P systems (1-step, 2-step, and multi-step) compared to Mylar strip is supported by many previous studies [21, 36, 39]. The lowest VMH noted with Mylar strip is explained by the high resin concentration of the outer layer, which results from oxygen inhibition beneath the celluloid strip and impairs the layer’s mechanical qualities.

Based on the results of this study, the first null hypothesis, which stated that the type of flowable bulk-fill resin composite had no significant effect on surface roughness, surface gloss, and microhardness was partially rejected. The results demonstrated that the type of FBRC significantly affected surface gloss and microhardness, but it had no significant effect on surface roughness. The results confirmed that the finishing and polishing techniques significantly affected surface roughness, surface gloss, and microhardness for all tested resin composites. Therefore, the second null hypothesis, which stated that the finishing and polishing technique had no significant effect on surface roughness, surface gloss, and microhardness of all tested flowable bulk-fill resin composites, was rejected. In this study, there was a significant correlation between surface roughness, gloss, and microhardness. The analysis showed a significant negative correlation between surface roughness and surface gloss. It can be attributed to smoother surface reflecting light more uniformly, enhancing gloss. A significant positive correlation was found between surface roughness and microhardness, and a significant negative correlation was observed between surface gloss and microhardness. It can be attributed to harder materials are more resistant to F/P procedures. Therefore, the third null hypothesis which stated that there was no correlation between surface roughness, surface gloss, and microhardness regarding the type of resin composite and the type of finishing and polishing system, was rejected.

Several limitations were identified during the study. The specimens were prepared and tested under ideal conditions. These conditions didn’t replicate the dynamic oral environment. Future studies should incorporate simulated oral environments (e.g., thermal cycling, mechanical loading, and pH variations) to assess long-term material behavior. For resin composite materials and finishing systems, only three types of FBRCs were included, and only three F/P systems were studied. So future studies should include more resin composite materials and finishing techniques to increase generalizability.

## Conclusions

Within the limitations of this study the following conclusions can be drawn:

1. It appeared that zirconium oxide fillers improved the flowable bulk-fill resin composites’ surface roughness, surface gloss, and microhardness.
2. Surface gloss and microhardness were significantly influenced by material type and polishing system used, while surface roughness was influenced only by polishing system used.
3. OptiDisc system achieved the smoothest and highest gloss surface with PALFIQUE BULK FLOW and Tetric N-Flow Bulk Fill, whereas the Sof-Lex Spiral system achieved the smoothest and highest gloss surface with Filtek Bulk Fill Flowable.
4. Compared to Mylar strip, all finishing and polishing systems used in this study enhanced the microhardness of all tested flowable bulk-fill resin composites.

## Data Availability

The data that support the findings of this study are available from the corresponding author upon reasonable request.

## Abbreviations

FBRCs: Flowable bulk-fill resin composites
F/P: Finishing and polishing
ZrO_2_: Zirconium oxide
PBF: PALFIQUE BULK FLOW
FBF: Filtek Bulk Fill Flowable
TBF: Tetric N-Flow Bulk Fill
SR: Surface roughness
SG: Surface gloss
VMH: Vickers microhardness
LED: Light-emitting diode
Ra: Arithmetic roughness mean
GU: Gloss unit
Bis-GMA: Bisphenol A glycerolate dimethacrylate
Bis-MPEPP: Bisphenol A polyethoxy dimethacrylate
TEGDMA: Triethylene glycol dimethacrylate
UV: Ultraviolet radiation
Bis-EMA: Ethoxylated bisphenol A glycol dimethacrylate
